# Study on orally delivered paclitaxel nanocrystals: modification, characterization and activity in the gastrointestinal tract

**DOI:** 10.1098/rsos.170753

**Published:** 2017-11-08

**Authors:** Runcong Liu, Ya-Nan Chang, Gengmei Xing, Min Li, Yuliang Zhao

**Affiliations:** CAS Key Laboratory for Biomedical Effects of Nanomaterial and Nanosafety, Institute of High Energy Physics, Chinese Academy of Sciences, 19B Yuquan Road, Shijingshan District, Beijing 100049, People's Republic of China

**Keywords:** oral delivery, paclitaxel nanocrystal, gastrointestinal tract

## Abstract

Drug nanocrystals (NCs) can improve the solubility and bioavailability of insoluble drugs for oral administration. However, the biocompatibility and mechanisms of transmittance of drug NCs through the intestinal epithelial tissue are still not well understood. In this work, the physico-chemical properties and interactions with biomolecules in oral delivery pathways, as well as the transmittance through mimical intestinal epithelial cells, of NCs of paclitaxel (PTX) are investigated. PTX was previously demonstrated to be an effective anti-cancer drug. It is found that maximum 1% (w/v) poly(styrenesulfonate) is sufficient to keep PTX NCs monodisperse in varied biological environments and presents no significant interaction with extracellular biomolecules for at least 24 h. The concentration of PTX NCs is kept carefully controlled to avoid serious toxicity to cells (10 µg ml^−1^ in our experiments but this also depends on NC size). The transmittance of PTX NCs through mimical intestinal epithelial reached 25% in 6 h, demonstrating its comparatively high oral bioavailability in the human body. This work demonstrates the great potential of PTX NC treated in oral delivery.

## 1. Introduction

Oral administration of drugs is generally the preferred route compared to the parenteral route due to its convenience, safety and reduced healthcare costs [1,2]. The oral route has also gained major focus and clinical recognition thanks to patient compliance. However, tough challenges still exist for oral delivery, for example, orally administrated drugs must be absorbed in the intestine to increase their bioavailability, but the physiological barriers of the gastrointestinal tract tend to prevent the absorption of many drugs, especially insoluble ones [3]. Particularly, proteolytic enzymes present throughout the gastrointestinal tract can interact with drugs and consequently decrease their efficacy [4]. Also, the low pH environment in the stomach due to gastric acid can degrade drugs [5]. Intestinal epithelium and its adherent mucus layer can also limit the permeation of drugs which possess high molecular weight or high polarity [6]. Thus, controlling the physico-chemical properties of drugs is the principal challenge to oral administration. Drug nanocrystals (NCs) (size in the approx. 100 nm range) can be formulated through ‘nanosuspension’ technology [7,8]. The goal of formulating NCs is to prolong the metabolism of drugs in the body, enhance their bioavailability, decrease the surface–volume ratio of drug and solve the problem of poor solubility [9–12]. Paclitaxel (PTX) is one of the most promising anti-tumour drugs, but it suffers from poor solubility [13,14]. PTX is currently administered by intravenous injection with a co-solvent which causes many problematic side effects (e.g. neurovirulence causing pain, acro-anaesthesia and high expense) [15–18]. PTX NCs have also been demonstrated to exhibit efficient anti-cancer activity [19,20] and are thus a highly desired candidate for oral administration. The investigation of the interactions between PTX NCs and biomolecules, especially enzymes, and their transmittance across the intestinal epithelium is of great interest for clinical applications [21–23].

Evaluation of the drug absorption potential is usually performed by a permeability assay, which is one of the most important criteria in the early stages of drug development [24]. Caco-2, a cell line derived from the human colon adenocarcinoma with remarkable morphological/biochemical similarities to the small intestinal epithelial cells of humans, has been widely used as an *in vitro* model for the prediction of intestinal drug permeation and absorption [25]. Caco-2 cells can slowly differentiate into a single-cell layer via tight junctions, affording the capability to selectively permeate certain molecules.

In this work, PTX NCs were prepared and studied for their oral delivery capability. In contrast with much of the investigation of the anti-cancer activity of PTX NCs, our work focuses less on discussion of the preparation and activity, but specifically on the delivery process, from oral administration until passage through the intestinal epithelial cells. The properties of PTX NCs in various simulated biological environments, their potential interactions with biomolecules, their bio-stability, toxicity and finally their transmission across mimical intestinal epithelium were carefully studied.

## 2. Material and methods

### 2.1. Materials

PTX NC was formulated by Zhang's group from Peking University. Hydroxypropyl methyl cellulose (HPMC), d-α-tocopherol polyethylene glycol 1000 succinate (TPGS) and poly(sodium-*p*-styrenesulfonate) (PSS), pepsin and trypsin were purchased from Sigma-Aldrich and used without further purification.

### 2.2. Atomic force microscope/scanning electron microscope characterization

PTX NCs were dispersed in purified water to a final concentration of 20 μg ml^−1^. Surfactant (5%), HCl (to pH = 2 to simulate the gastric acid environment) or specific enzymes (pepsase and trypsase) were added depending on the experiment. The above solution was sonicated for 20** **min to separate aggregated PTX NC. Then a droplet of the solution was deposited on a clean flat silicon surface and let sit for 5** **min, the surface was then dried with a gentle nitrogen flow before being imaged with either a Bruker atomic force microscope (AFM) or Hitachi scanning electron microscope (SEM). The surface was coated with gold prior to SEM imaging.

### 2.3. Dynamic light scattering/zeta-potential measurement

PTX NCs were dispersed in purified water to a final concentration of 20 μg ml^−1^. Surfactant (5% in w/v) and HCl (to pH = 2 to simulate the gastric acid environment or specific enzymes) were added. The particle size and zeta-potential were then determined by a Nicomp380 dynamic light scattering (DLS) plus ZETA system.

### 2.4. Paclitaxel nanocrystal–enzyme interaction measurement

Enzymes were dissolved in aqueous solution (HCl with pH = 2 for pepsin and PBS with pH = 7 for trypsin) to a final concentration of 1** **mg ml^−1^. Surfactants were added to the enzyme solution to the same final concentration as that used for PTX NC modification (0.25% in w/v). The absorbance of the above solutions was determined by UV–vis spectrophotometer. Then, 50 μl of PTX NC solution with a concentration of 1** **mg µl^−1^ was incubated with the above solution (2** **ml) for 1** **h. Afterwards, the solution was filtered with a pinhole membrane (0.22 μm in diameter for the nanohole) to remove PTX NC and PTX NC–enzyme (if formed) and its absorbance spectrum determined again. The intensity of the absorbance at 290** **nm was analysed to determine the interaction between enzymes and PTX NCs.

### 2.5. Cytotoxicity study of paclitaxel nanocrystal

Caco-2 cell lines (human colon carcinoma) were employed to simulate the intestinal epithelial system. Cells were grown at 37°C and 5% CO_2_ in a constant humidity environment in minimal essential medium (MEM, Gibco, Invitrogen) containing glucose and non-essential amino acids (1%, NEAA, Gibco, Invitrogen), 50 U ml^−1^ penicillin and 50 μg ml^−1^ streptomycin, HEPES (10** **mM) and 20% fetal calf serum, and the growth medium was changed every other day until use. For the toxicity test, the cells were distributed to 96-well plates (10^4^ cells well^−1^) for groups (*N* > 4 for each group), the medium of each group was MEM with different concentrations of PTX NCs. After incubating the cells at 37°C for a set amount of time (24 or 48 h), the cell viability of each group was determined to estimate the toxicity of PTX NCs. Cell viability assays were performed using a Cell Counting-8 Kit (CCK-8, Dojindo Laboratories, Japan) by following the manufacturer's instructions.

### 2.6. Transmittance of paclitaxel nanocrystal through Caco-2 cell monolayers

Caco-2 cells were seeded in 24-well transwell plates at a density of 10^5^ cells well^−1^. The Caco-2 cells were allowed to attach and grow in culture medium for 21 days, and the medium was refreshed every 2 days. At day 21, caco-2 monolayers were formed, and treated with PTX NC (10 μg ml^−1^) at 37°C for 0** **min, 5** **min, 10** **min, 30** **min, 1** **h, 4** **h and 6** **h. At each time point, the HBSS buffer was collected in the lower chamber. The experiments were duplicated three times independently. Absorbance spectra of aqueous suspensions of PTX NCs were recorded at 290** **nm on a UV–vis spectrometer (Persee General, Beijing, China). PTX NC transmittance was normalized with a buffer sample as a blank reference.

## 3. Results and discussion

### 3.1. Size variation and surface modification of paclitaxel nanocrystals

**Figure 1.**
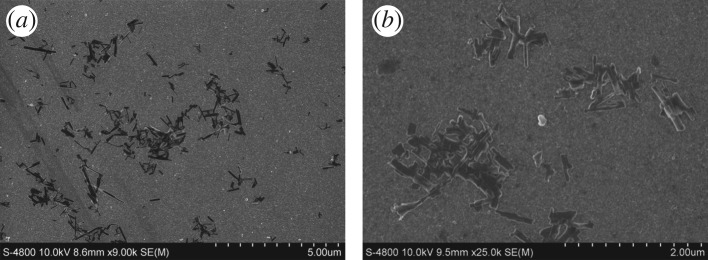
Large- (*a*) and small-scale (*b*) SEM image of unmodified PTX NC on Si(100) surface.

**Figure 2.**
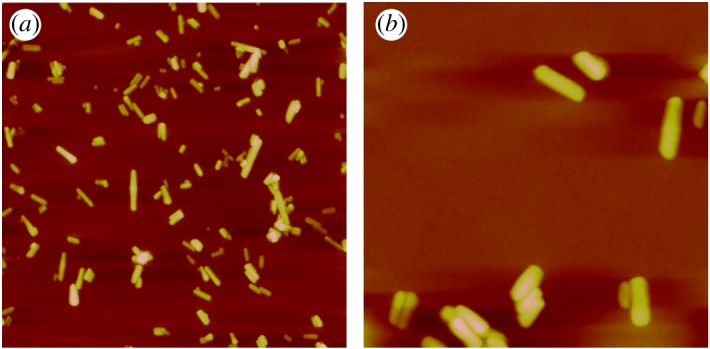
(*a*) The 20 µm × 20 µm and (*b*) 4 µm × 4 µm AFM images of PSS-modified PTX NC on mica.

Size is a critical parameter for the oral delivery of drug NCs. Endocytosis, which is an important way for drugs to cross intestinal epithelial cells, is sensitive to the size and geometry of the nanoparticle. Owing to the poor solubility of PTX NCs, unmodified PTX NCs tend to aggregate and may even coagulate in aqueous environments, which can be clearly observed from the SEM images (figure 1). DLS was employed to evaluate the aggregation state of the NCs. The average particle size was measured to be 1.3 μm with polydispersity index (PDI) approximately 0.4 in both acidic (pH = 2) and neutral environment. In addition, the zeta-potential of the PTX NCs in neutral environment was found to be −8** **mV. As the size of aggregated PTX NCs is similar to that of a cell, it makes PTX NCs difficult to pass through the intestinal epithelial cells and thus decreases PTX NC efficacy. An individual NC could be a more effective oral drug if its solubility was improved. HPMC, TPGS and PSS were thus introduced here as surfactants on the PTX NC surface due to their low biological toxicity [22]. We employed AFM to monitor the size and geometry of the formulated PTX NCs after PSS modification. The results are presented in figure 2. It is clearly seen that PTX NCs present as quasi-monodisperse after modification. PTX NCs have rod-like shapes with different lengths. The average length of individual PTX NCs is 320 ± 40** **nm, and the diameter was determined to be 30 ± 5** **nm. Clearly, the size of individual PTX NCs could be controlled by surfactant to meet the size requirements of cell endocytosis (less than 1 μm) [26,27].

**Figure 3.**
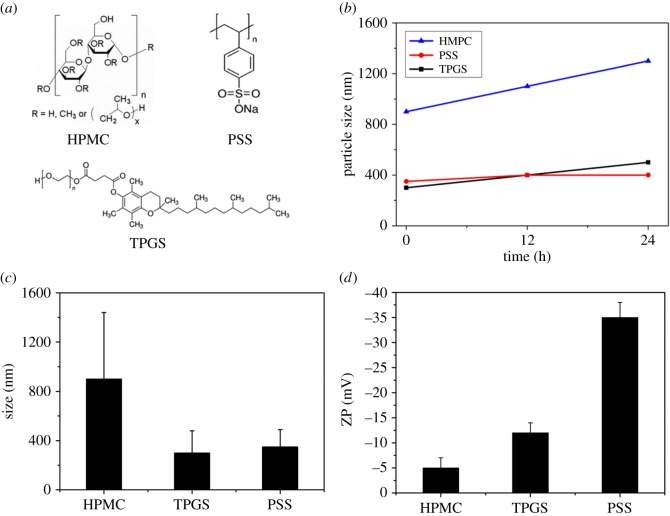
(*a*) Chemical structure of the surfactants. (*b*) Stability of different surfactant-modified PTX NC with time at pH = 7. DLS (*c*) and zeta-potential (*d*) of PTX NC modified with different surfactants at pH = 7.

The size of PSS-, TPGS- and HPMC-modified PTX NCs were determined by DLS to be 300** **nm, 350** **nm and 900** **nm, respectively. Clearly, the size of PSS- or TPGS-modified PTX NCs is close to that of an individual NC, indicating the monodispersion of NCs after modification (figure 3). While TPGS-modified PTX NCs present the smallest particle sizes shortly after incubation, PSS-modified PTX NCs show the most colloidal stability and least change over time, as indicated in figure 3*b*, which is also in good agreement with the zeta-potential value (−35** **mV) for PSS-modified PTX NCs (figure 3*c*,*d*). TPGS modification leads to a smaller particle size (300** **nm), the PDI value (0.35) is, however, larger than that for PSS, indicating more variation in particle size caused by PTX NC aggregation. The zeta-potential of TPGS-modified PTX NCs is −12** **mV, suggesting they are less stable than PSS-modified PTX NCs (−35** **mV). PSS is thus considered preferential among the three surfactants, and PSS-modified PTX NCs were chosen as the candidate in the following experiments.

### 3.2. Stability of paclitaxel nanocrystal in gastrointestinal environments

**Figure 4.**
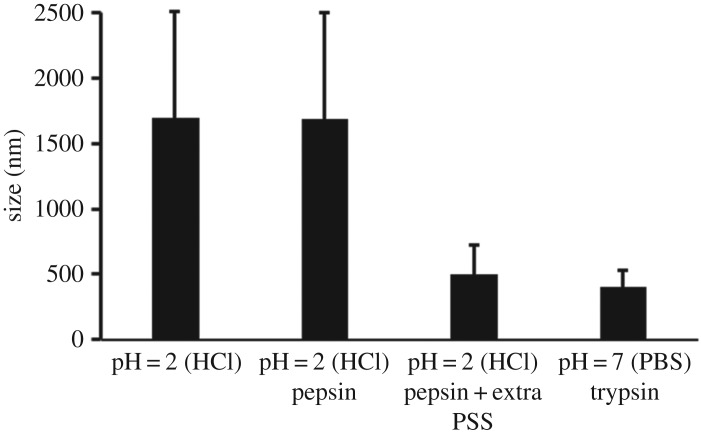
Particle size dependence of PSS-modified PTX NC on pH value and the present enzyme in solution.

PTX NCs need to pass through various biological environments before reaching the intestinal epithelium. Though formulating PTX into NCs improved its solubility, the varied conditions and biological molecules present in the gastrointestinal tract could change the properties of PTX NCs. We first examined if the enzymes in gastrointestinal systems would interact with PTX NCs considering these enzymes are known for digestion. PTX NCs were observed to aggregate in simulated gastric environment (HCl, pH = 2 with pepsin) with particle size dramatically increasing to 1700 nm. Interestingly, removing the enzyme pepsin does not cause any significant change in the particle size, suggesting the aggregation has little correlation with pepsin, as shown in figure 4. This indicates that pH value could be the principal factor leading to PTX NC aggregation. This was further confirmed in parallel experiments in simulated intestinal environment showing that PTX NCs present good monodispersions in PBS solution with pH = 7. Interestingly, the aggregation problem can be partially solved by adding more PSS. It should be noted that such aggregation is not reversible: changing pH from approximately 2 to approximately 7 will not re-disperse PTX NCs. This could cause trouble if the PTX NC is designed for oral delivery. Our results show that increasing the concentration of surfactants (to approx. 1% in w/v) can largely prevent PTX NC aggregation and improve its colloidal stability, especially in gastric conditions.

### 3.3. Interactions of paclitaxel nanocrystal with pepsin/trypsin

It has been reported that biomolecules present in biological systems tend to adsorb onto the surface of exotic nanoparticles to form a ‘corona’ [24,28]. The corona will modify the particle surface helping the biological system to treat the particle in a specific way. For nano-drugs adopting the oral delivery pathway, bio-adsorption on the drug particles will no doubt alter their surface properties and further influence their efficacy. Therefore, the adsorption of biomolecules onto nano-drugs is a serious issue and should be taken into consideration when studying the downstream behaviour of nano-drugs. We take pepsin and trypsin as two examples to study bio-molecular adsorption onto orally administered PTX NC.

**Figure 5.**
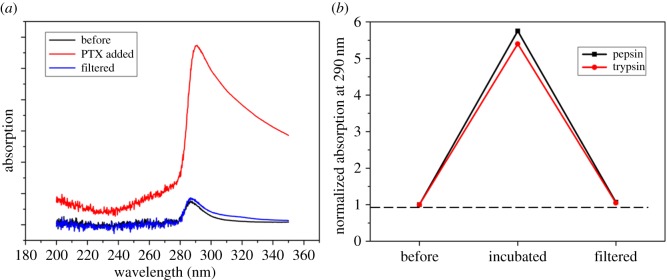
(*a*) Absorption tendency and (*b*) normalized absorption at approximately 290 nm of pepsin/trypsin before and after incubation with PTX NC.

The absorbance spectra of pepsin and trypsin before and after incubation with PTX NC are shown in figure 5. Thanks to the significant difference in sizes between NC and pepsin/trypsin, they can be separated easily by filtering. If there is an adsorption, pepsin/trypsin-attached NCs should be blocked by the nanoholes in the pinhole membrane and lead to a concentration/absorbance drop.

Figure 5 shows that the absorbance at approximately 290** **nm increased pronouncedly after pepsin/trypsin was incubated with PTX NCs, which is attributed to the electronic transition in the phenyl ring group present in both enzymes and PTX. After filtering, the absorbance value returns close to the original level but remains slightly higher than that before PTX NC was added as indicated by the dotted line (figure 5*b*). The slight increase in the absorbance value was caused by the presence of a small amount of PTX in solution, which was confirmed by comparing it with the absorbance value of filtered pure PTX NCs (a weak signal at approx. 290** **nm appeared due to some PTX NCs present in solution passing through the pinhole membrane). These results indicate that neither pepsin nor trypsin has significant interaction with PSS-modified PTX NCs.

### 3.4. Toxicity of paclitaxel nanocrystal to intestinal epithelial cell

Orally administrated drugs have to pass through the intestinal epithelial cells and then enter blood circulation. The intestinal epithelial cell could be damaged during this process if the drug is toxic. Drug toxicity is complicated and dependent on many factors such as size, concentration and retention time in the human body. Here, we checked the toxicity of PTX NCs by monitoring cell viability after exposure of cells to PTX NC solutions with different concentrations for 24 h and 48 h, respectively. The goal of this test was to determine a safe concentration at which the orally delivered PTX shows no significant toxicity to intestinal epithelial cells. It has been reported that currently only a small part (usually around 10% or lower) of orally delivered NCs can be taken up by the human body [16]. Most NCs remain in the intestinal system for several more hours. Based on this, 24** **h was selected as the time scale for testing cell viability upon exposure to PTX NCs.

**Figure 6.**
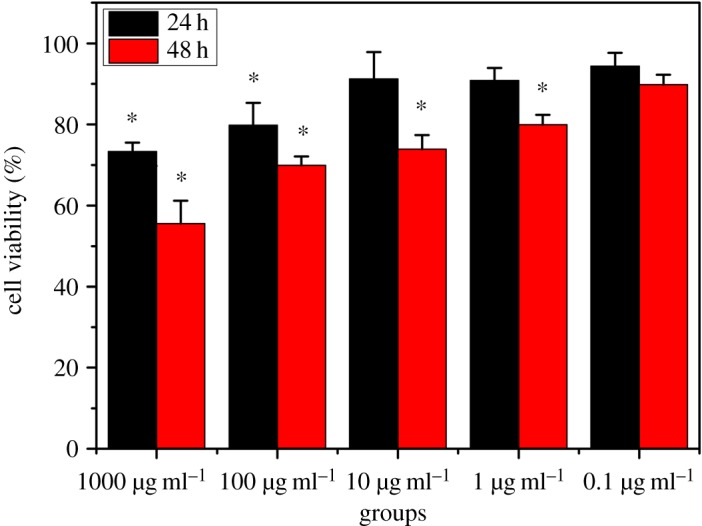
Toxicity of PTX NC under different concentration and time conditions. *p < 0.05.

Figure 6 shows that upon 24 h exposure to PTX NCs, most of the cells (greater than 90%) survive at low PTX NC concentration (10 μg ml^−1^). The viability of cells decreased from 90 to 73% after incubation with PTX NCs for 48** **h at low PTX NC concentrations 0.1–10 μg ml^−1^. At higher concentration, PTX NCs presents significant toxicity, cell viability reducing to 70 and 56% in the case of 100 and 1000 μg ml^−1^ PTX NC exposure, respectively. Accordingly, the amount of orally administered PTX NCs should be carefully controlled to avoid significant toxicity to the human body, especially to the intestinal cells.

### 3.5. Transmittance of paclitaxel nanocrystal through mimical intestinal epithelial cells

**Figure 7.**
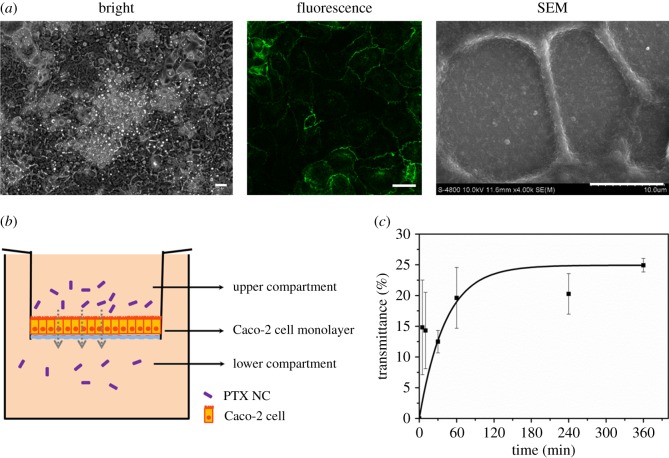
Transmittance of PTX NC over monolayer cell. (*a*) Bright field microscope, fluorescence microscope and SEM imaging of Caco-2 cell monolayer after being cultured for 21 days. The green fluorescence shows the tight junction protein expression. All bars in the picture are 10 µm. (*b*) Schematic diagram of PTX NC passing through the mimical intestinal epithelial cell. (*c*) Transmittance of PTX NC over the mimical intestinal epithelial cell at 0 min, 5 min, 10 min, 30 min, 1 h, 4 h and 6 h. The black solid line is a fit to an asymptotic curve (see the text).

Transmittance studies were performed in pH 7.4 HBSS medium, in which Caco-2 cells grew on the upper chamber of the transwell for 21 days to finally form the mimic intestinal epithelial cell monolayer. The formed Caco-2 cell monolayer was imaged by bright field microscopy, fluorescence microscopy and SEM, as shown in figure 7*a*. The immunofluorescence and SEM images clearly show the tight junction existing between cells, suggesting the successful formation of a Caco-2 cell monolayer. It is reported that PTX NCs mainly transport in a transcytosis manner rather than a paracellular pathway on cell monolayers [29]. The schematic diagram of PTX NCs passing through the mimical intestinal epithelial cell is shown in figure 7*b*. In order to evaluate the bioavailability of orally delivered PTX NCs by the human body, the transmittance of PTX NCs through mimical intestinal epithelial cells was measured with UV–vis absorption spectroscopy, and the results are shown in figure 7*c*. The black solid line is a fit to an asymptotic equation (*T* = −24.929*e*^(−^*^t^*^)/41.94^ + 24.929, where *T* is the transmittance of PTX NC, *t* is time, *e* is the natural constant, approx. equal to 2.718) in data processing. It is speculated that ingested nanoparticles need several hours, normally 4–8** **h, to reach the gut intestine tract and interact with epithelial cells after oral administration [30]. To understand the time dependence of PTX NC transmittance, 5** **min, 10** **min, 30** **min, 1** **h, 4** **h and 6** **h transmembrane PTX NC transmission was investigated. About 10–15% of PTX NCs passed through the cell membrane within 10** **min, and the transmittance reached over 25% in 6** **h as shown in figure 7*c*. The pronounced change in the transmittance occurred within 1** **h and then reached a plateau after 1** **h, which is in good agreement with previously reported results [31,32]. In our work, it is found that 6 h is a critical time scale for the drug to be taken up by the intestinal systems. It should be noted that the concentration of PTX NCs in the transmittance experiments was determined to be approximately 10 μg ml^−1^ to ensure reduced toxicity. The transmittance experiments demonstrate both the capability of PSS-modified PTX NCs to penetrate into the intestinal epithelium and their comparatively high oral bioavailability for the human body, which has rarely been reported elsewhere.

## 4. Conclusion

The interactions and interference of PTX NCs with biomolecules in the oral delivery pathway were investigated. PSS-modified PTX NCs present good monodispersion and stability in gastrointestinal environments for at least 24 h, which is both sufficient and necessary for oral delivery. No significant interactions between PSS-modified PTX NCs and pepsin/trypsin were observed in gastrointestinal tract environments, suggesting PTX NCs' ability to pass through gastrointestinal tracts with little interference to intestinal epithelial cells. Owing to their toxicity to cells, PTX NCs should be limited to a maximum concentration of around 10 μg ml^−1^, for which PSS-modified PTX NCs with proper geometry (rod-like shape, approx. 300** **nm in length and approx. 30 nm in diameter) can pass through the mimical intestinal epithelial cell with a transmittance of approximately 25%, presenting a comparatively high oral bioavailability for the human body. Worthy of note is that the safe concentration of PTX NCs is related to the size of the NCs. These results demonstrate the great potential of PSS-modified PTX NCs as anti-cancer drugs for oral administration. This work serves as a preliminary support for practical applications of NCs in the oral delivery pathway.

## Supplementary Material

Supplimentary Material from “Study on Orally Delivered Paclitaxel Nanocrystals: Modification, Characterization and Activity in the Gastrointestinal Tract”

## Data accessibility

The datasets supporting this article are found in the electronic supplementary material.
