# Supplementary material for: Study on orally delivered paclitaxel nanocrystals: modification, characterization and activity in the gastrointestinal tract
Source: R Soc Open Sci. 2017 Nov 8;4(11):170753. doi: 10.1098/rsos.170753 (PMC5717641; doi:10.1098/rsos.170753)
Supplement: Supplimentary Material from “Study on Orally Delivered Paclitaxel Nanocrystals: Modification, Characterization and Activity in the Gastrointestinal Tract” [file rsos170753supp1.docx]

Supplimentary Material

of

Study on Orally Delivered Paclitaxel Nanocrystals: Modification, Characterization and Activity in the Gastrointestinal Tract

Runcong Liu^†^, Ya-Nan Chang^†^, Gengmei Xing, Min Li^*^, Yuliang Zhao

*CAS Key Laboratory for Biomedical Effects of Nanomaterial and Nanosafety, Institute of High Energy Physics, Chinese Academy of Sciences, Beijing, 100049, China*


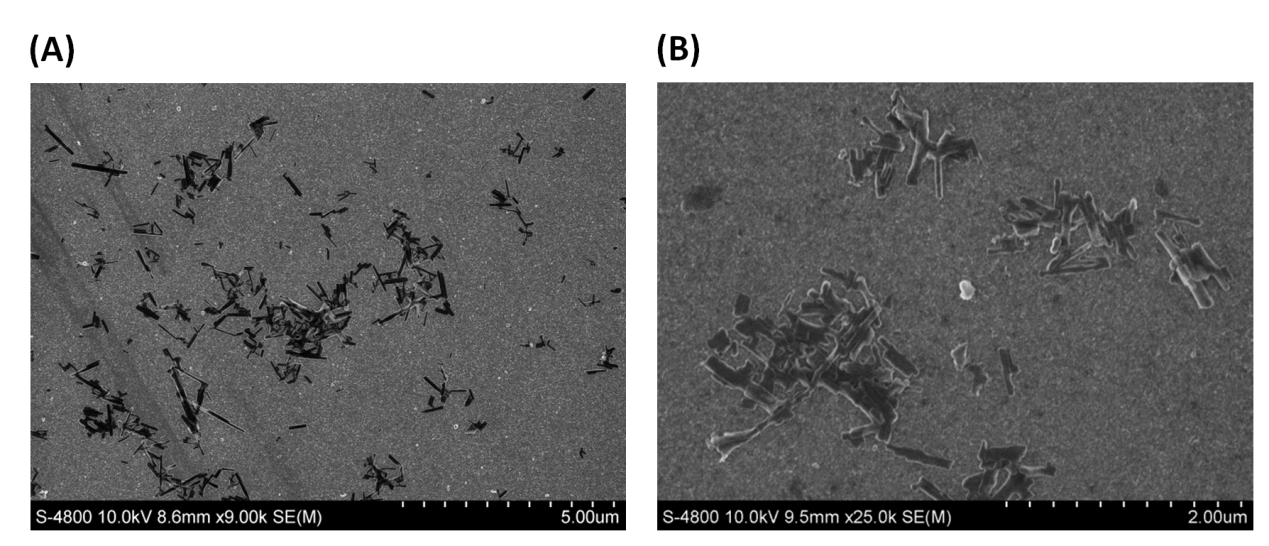


Figure 1 Large- (A) and small- (B) scale SEM image of unmodified PTX NC on Si(100) surface.


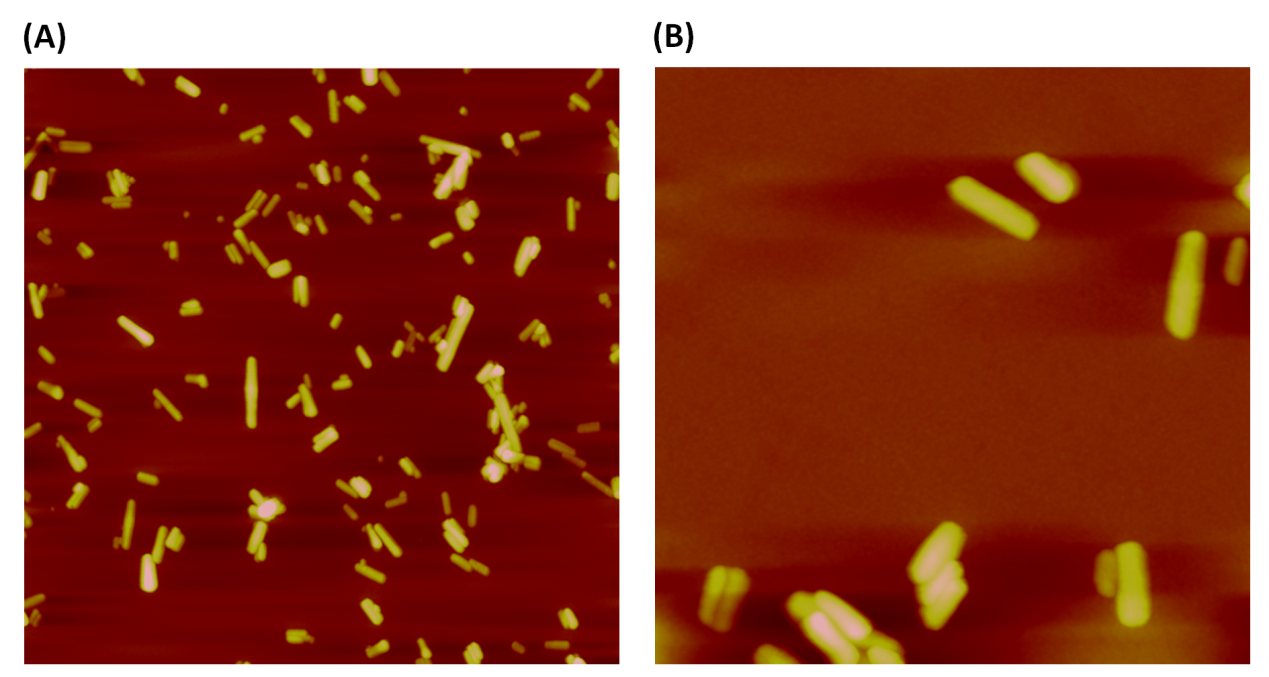


Figure 2 (A) 20 um × 20 um and (B) 4 um × 4 um AFM images of PSS modified PTX NC on mica.


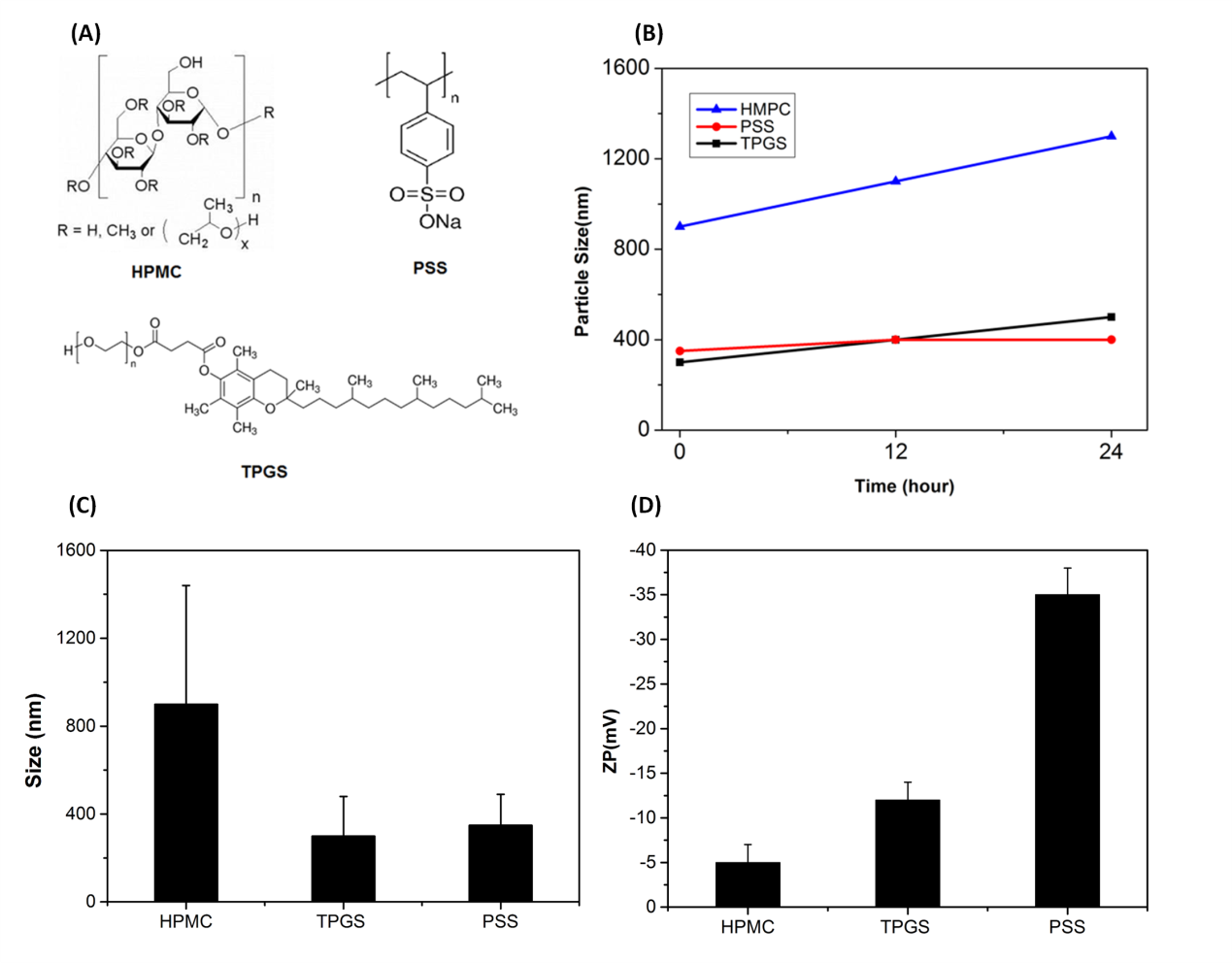


Figure 3 (A) Chemical structure of the surfactants. (B) Stability of different surfactant modified PTX NC with time at pH = 7. DLS (C) and Zeta-potential (D) of PTX NC modified with different surfactants at pH = 7.


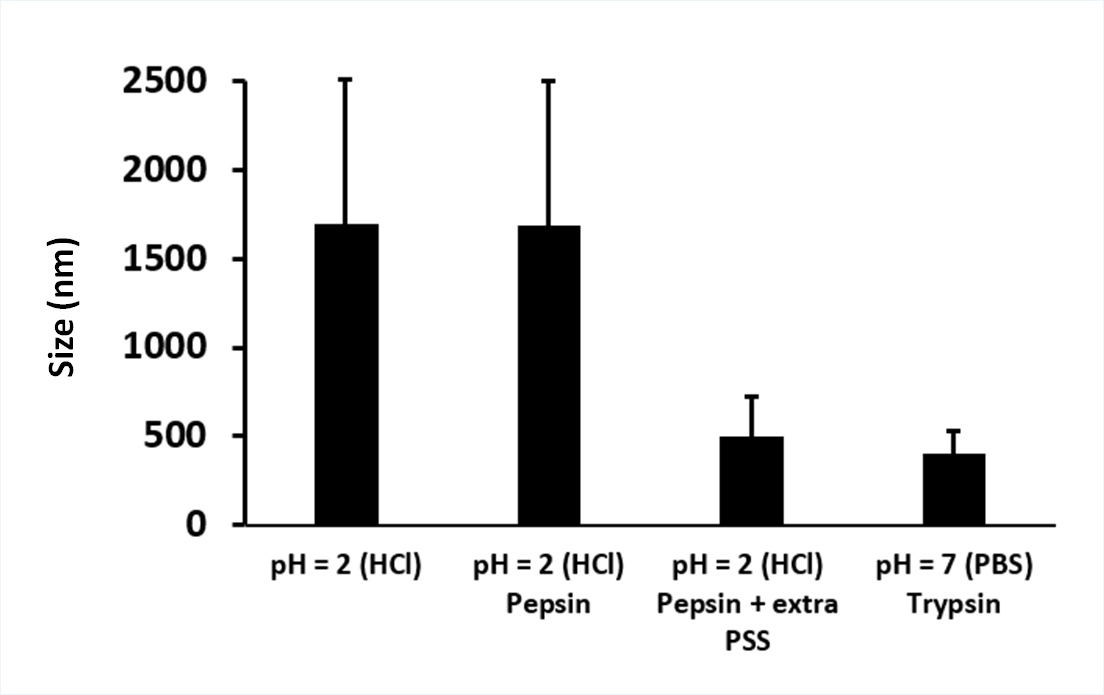


Figure 4 Particle size dependence of PSS modified PTX NC on pH value and the present enzyme in solution.


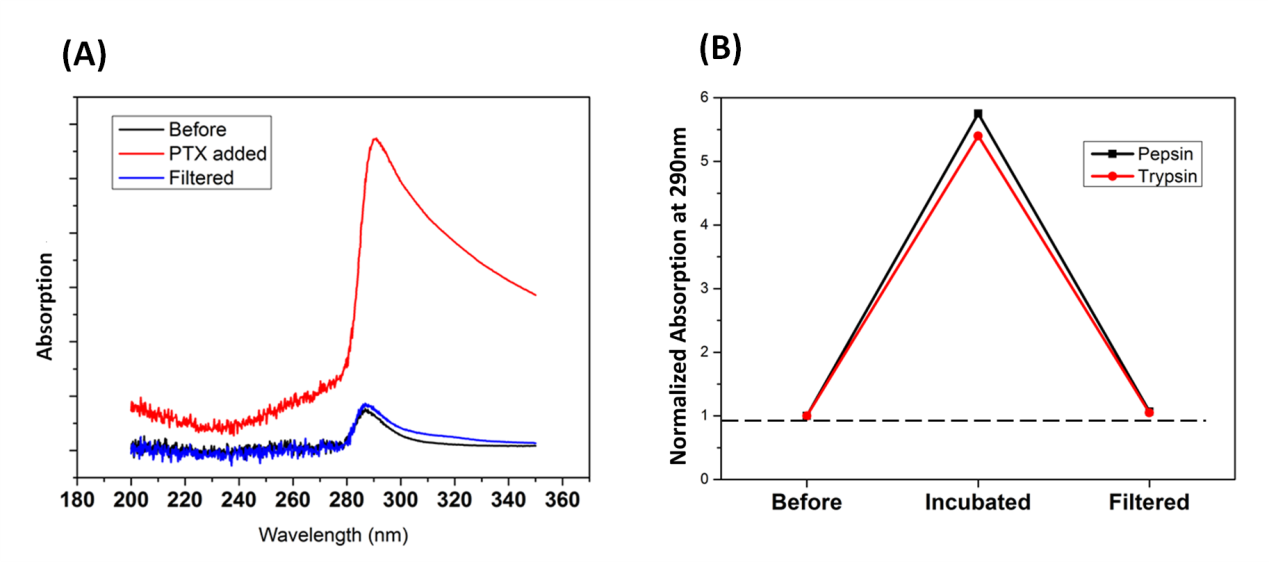


Figure 5 (A) Absorption tendency and (B) Normalized absorption at ~290nm of pepsin/trypsin before and after incubation with PTX NC





Figure 6 Toxicity of PTX NC under different concentration and time conditions. **p<0.05*


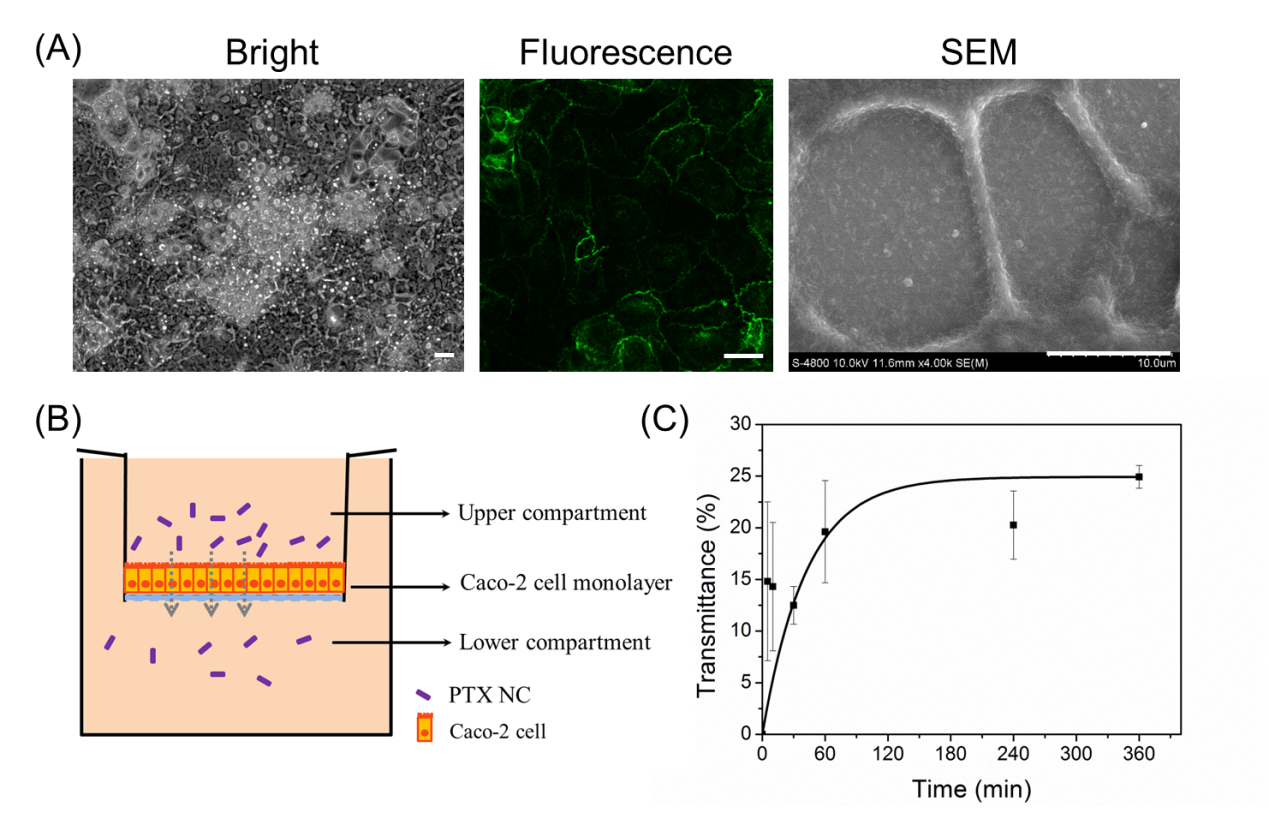


Figure 7 Transmittance of PTX NC over monolayer cell. (A) Bright field microscope, fluorescence microscope and SEM imaging of Caco-2 cell monolayer after cultured for 21 days. The green fluorescence shows the tight junction protein expression. All bars in the picture are 10 μm. (B) Schematic diagram of PTX NC passing through the mimical intestinal epithelial cell. (C) Transmittance of PTX NC over the mimical intestinal epithelial cell at 0 min, 5 min, 10 min, 30 min, 1h, 4h and 6h. The black solid line is a fit to an asymptotic curve (see the text).
